# Genetic lineage tracing reveals stellate cells as contributors to myofibroblasts in pancreas and islet fibrosis

**DOI:** 10.1016/j.isci.2023.106988

**Published:** 2023-05-26

**Authors:** Jinbang Wang, Tingting Li, Yunting Zhou, Xiaohang Wang, Vladmir Carvalho, Chengming Ni, Wei Li, Qianqian Wang, Yang Chen, Zhanjia Shang, Shanhu Qiu, Zilin Sun

**Affiliations:** 1 Department of Endocrinology, Zhongda Hospital, Institute of Diabetes, School of Medicine, Southeast University, Nanjing, China; 2 Department of General Practice, Zhongda Hospital, Institute of Diabetes, School of Medicine, Southeast University, Nanjing, China; 3 Department of Endocrinology, Nanjing First Hospital, Nanjing Medical University, Nanjing, Jiangsu, China; 4 Department of Endocrinology, Suzhou Hospital of Anhui Medical University, Suzhou, China

**Keywords:** Endocrinology, Cell biology

## Abstract

Pancreatic stellate cells (PSCs) are suggested to play an important role in the development of pancreas and islet fibrosis. However, the precise contributions and solid *in vivo* evidence of PSCs to the fibrogenesis remain to be elucidated. Here, we developed a novel fate-tracing strategy for PSCs by vitamin A administration in Lrat-cre; Rosa26-tdTomato transgenic mouse. The results showed that stellate cells give rise to 65.7% of myofibroblasts in cerulein-induced pancreatic exocrine fibrosis. In addition, stellate cells in islets increase and contribute partly to myofibroblasts pool in streptozocin-induced acute or chronic islet injury and fibrosis. Furthermore, we substantiated the functional contribution of PSCs to fibrogenesis of pancreatic exocrine and islet in PSCs ablated mice. We also found stellate cells’ genetic ablation can improve pancreatic exocrine but not islet fibrosis. Together, our data indicates that stellate cells are vital/partial contributors to myofibroblasts in pancreatic exocrine/islet fibrosis.

## Highlights

• PSCs are traced successfully in Lrat Cre; Rosa26-tdTomato mice
• PSCs are vital contributors to the myofibroblast in pancreatic fibrosis
• PSCs in islet contribute to the myofibroblast pool in islet fibrosis
• PSCs genetic ablation improves pancreatic exocrine but not islet fibrosis

## Graphical abstract

**Figure:**
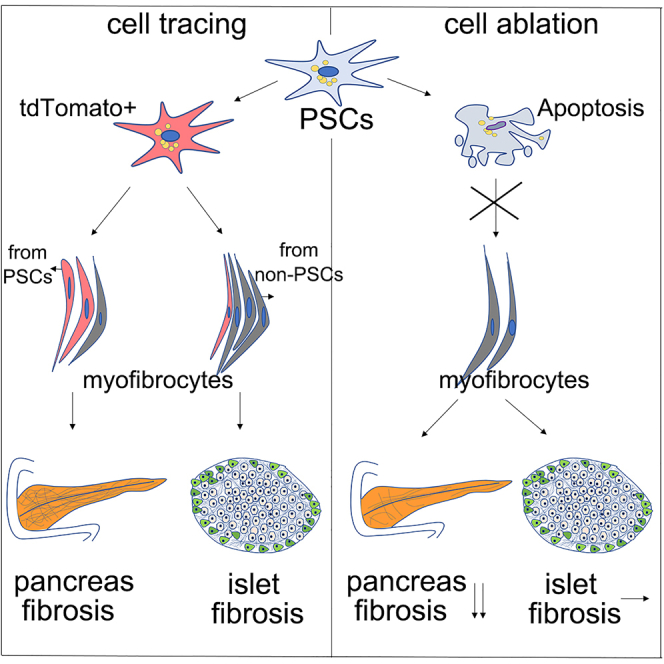

## Introduction

Fibrosis is a common pathological outcome of several etiological conditions, resulting in organ dysfunction and eventual failure.^1^ Excessive accumulation of extracellular matrix (ECM) components is a major feature of fibrosis.^2^ In addition, current evidences show that myofibroblasts are key players in ECM synthesis and can be derived from several cell types in organ fibrogenesis.^3^ Hepatic/pancreatic stellate cells, pericytes, fibroblasts, and endothelial cells (Endo-MT) have all been suggested as contributors to the myofibroblast pool.^3^^,^^4^

Pancreatic fibrosis has been recognized as a key determinant of the pathogenesis and progression of chronic pancreatitis and pancreatic cancer.^5^ Likewise, endocrine islet fibrosis in diabetes may affect insulin secretion and accelerate disease progression.^6^^,^^7^ Pancreatic stellate cells (PSCs), firstly discovered by Watari et al. in 1982, are traditionally considered as pancreatic counterpart cells of hepatic stellate cells.^8^ In 1998, two groundbreaking reports described the separation, culture, and characteristic expression of this type of cell, significantly promoting PSCs research progress.^9^^,^^10^ Then most subsequent studies focused on PSCs to explore the pathogenesis and therapeutic strategies of pancreatic fibrosis despite the lack of *in vivo* evidence.^11^ Besides, PSCs may migrate into islets and participate in the fibrotic process.^12^ However, little progress has been made to establish and precisely quantify the relative contribution of PSCs to the pancreatic myofibroblast pool and pancreatic fibrosis *in vivo*.

Vitamin A (VA) storage is the main physiological function of HSCs/PSCs.^13^^,^^14^ In addition, Lecithin:retinol acyltransferase (LRAT) is the key enzyme responsible for retinyl-esters formation from retinol in HSCs/PSCs.^15^ Previous studies employing Cre driven by Lrat promoter have traced HSCs successfully.^4^ Here, we develop a novel fate-tracing strategy for stellate cells by VA administration in Lrat-cre transgenic mice. In addition, we demonstrated that stellate cells are the dominant contributors to collagen-producing myofibroblasts in cerulein-induced pancreatic exocrine fibrosis, and partial participants in islet fibrosis induced by streptozocin (STZ). Furthermore, we substantiated the functional contribution of stellate cells to pancreatic fibrogenesis in genetic cell ablation mice. Consistently, we found that stellate cells’ genetic ablation can improve pancreatic exocrine fibrosis.

## Results

### Lrat Cre labels pancreatic stellate cells gradually when mice are intragastrically loaded with vitamin A palmitate

**Figure 1:**
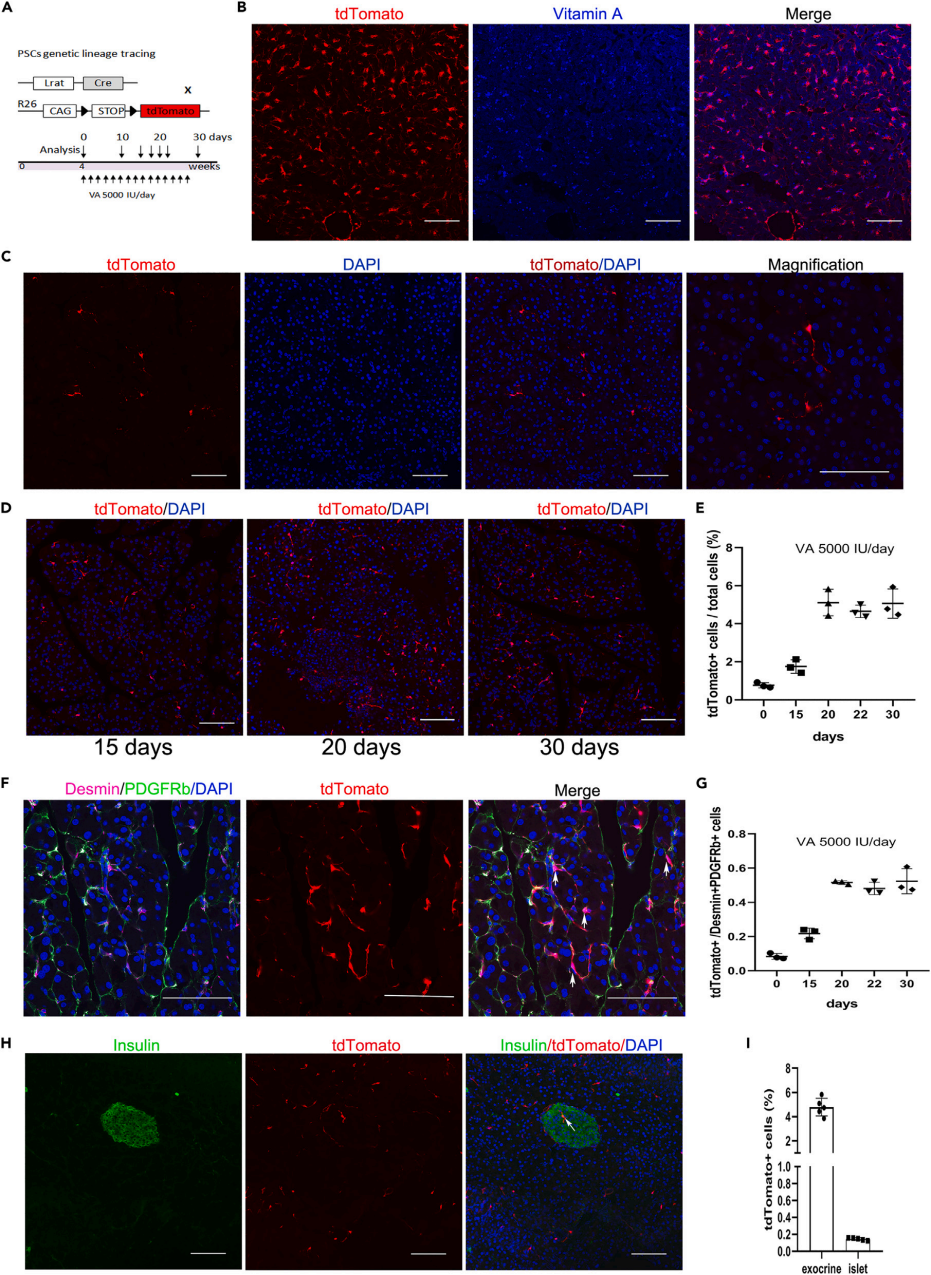
Lrat Cre labels stellate cells gradually when mice are intragastrically loaded with vitamin A (A) Schematic diagram illustrating the generation of genetic lineage tracing of stellate cells and the experimental strategy. Male mice aged 4 weeks were treated with vitamin A palmitate (5000 IU/day) for 0, 10, 15, 20, or 30 days and then euthanized at 3 days after the last dose. (B) Co-localization of Lrat Cre induced tdTomato with vitamin A autofluorescence (stellate cell marker) in the liver of mice without vitamin A palmitate gavage. (C) A few tdTomato+ cells in the pancreas of Lrat Cre mice without vitamin A administration. (D and E) tdTomato+ cells gradually increased (before 20 days) and reached a certain level (after 20 days) in the pancreas of Lrat Cre mice intragastrically loaded with vitamin A (5000 IU/day). (F and G) Co-localization of Desmin, PDGFRb with LratCre-induced tdTomato in pancreas and quantification of Lrat Cre-labelled tdTomato+ cells in Desmin+PDGFRb+ cells. (H and I) Quantification for tdTomato+ stellate cells in the exocrine pancreas or the islets. Scale bars, 100 μm. Data are shown as means ± s.e.

The storage of vitamin A (retinol) is the main physiological function of the stellate cell system.^16^ Retinol is esterified by Lecithin retinol acyltransferase (LRAT, the key enzyme responsible for retinyl ester synthesis) and then stored in stellate cells.^15^^,^^17^^,^^18^^,^^19^ To genetically label stellate cells, we crossed Lrat Cre mice with Rosa26-LSL-tdTomato reporter mice (Figure 1A). Lrat Cre transgenic mice marked hepatic stellate cells (HSCs) well as demonstrated by the nearly complete overlap of vitamin A autofluorescence (HSCs marker) with Lrat Cre-induced tdTomato reporter by confocal microscopy (Figure 1B). However, only few PSCs were labeled with tdTomato reporter in Lrat Cre transgenic mice (Figure 1C). This may be because of the fact that vitamin A is primarily stored in HSCs rather than PSCs.^18^^,^^20^ (Figure S1A). Therefore, we tried to improve the labeling efficiency based on vitamin A anabolic function of LRAT protein.^18^^,^^19^ We up-regulated the transcription of Lrat gene, thereby increasing Cre expression by Vitamin A loading (5000 IU/day). In addition, the number of tdTomato labeled cells in pancreas gradually increased (before 20 days) and reached stability (after 20 days) when mice were intragastrically loaded with vitamin A palmitate (Figures 1D and 1E). We also found that vitamin A accumulation in PSC in mice treated with vitamin A palmitate (Figure S1B). The increase of marked PSC was not because of PSC proliferation as demonstrated by the nearly no overlap of ki67 with tdTomato (Figure S1C). The tdTomato-labeled cells were stellate cells as demonstrated by immunofluorescence, showing nearly all the tdTomato+ cells expressed stellate cell markers (desmin and PDGFRb) by confocal microscopy (Figures S2A and S2B). The percentage of tdTomato+ cells in Desmin+PDGFRb+ cells increased (before 20 days) and reached stability (after 20 days) when mice were intragastrically loaded with vitamin A palmitate (Figures 1F and 1G). Desmin and PDGFRb are also expressed in pericytes, so we evaluated the proportion of stellate cells or pericytes in the pancreas by staining for Crbp1 (a stellate cell marker) and NG2 (pericytes marker) (Figures S2C and S2D). The proportion of labeled cells was similar to the proportion of actual stellate cells in total cells, so we believed that 20-day vitamin A administration could obtain a high stellate cell labeling efficiency. Pancreatic stellate cells were mainly distributed in the exocrine pancreas, whereas a small portion was observed in the islets, as demonstrated by the tdTomato+ cells accounting for 4.78 ± 0.74% of the total cells in the exocrine part and 0.14 ± 0.015% of total cells in islets (Figures 1H and 1I). To exclude the possible influence of bone marrow-derived cells to our tracing mice, we examined the bone marrow smears and bone marrow tissue from our cell tracing mice, and found no cells were labeled with tdTomato fluorescence (Figures S2E and S2F). This implies that the stellate cell which we focused on in this study was not affected by BM-derived cell in our tracing mice. Therefore, by vitamin A administration for 20 days, we developed a novel fate-tracing method for stellate cells in the Lrat Cre; Rosa26-tdTomato mice.

### Stellate cells are vital contributors to the myofibroblast pool in cerulein-induced pancreatic exocrine fibrosis

**Figure 2:**
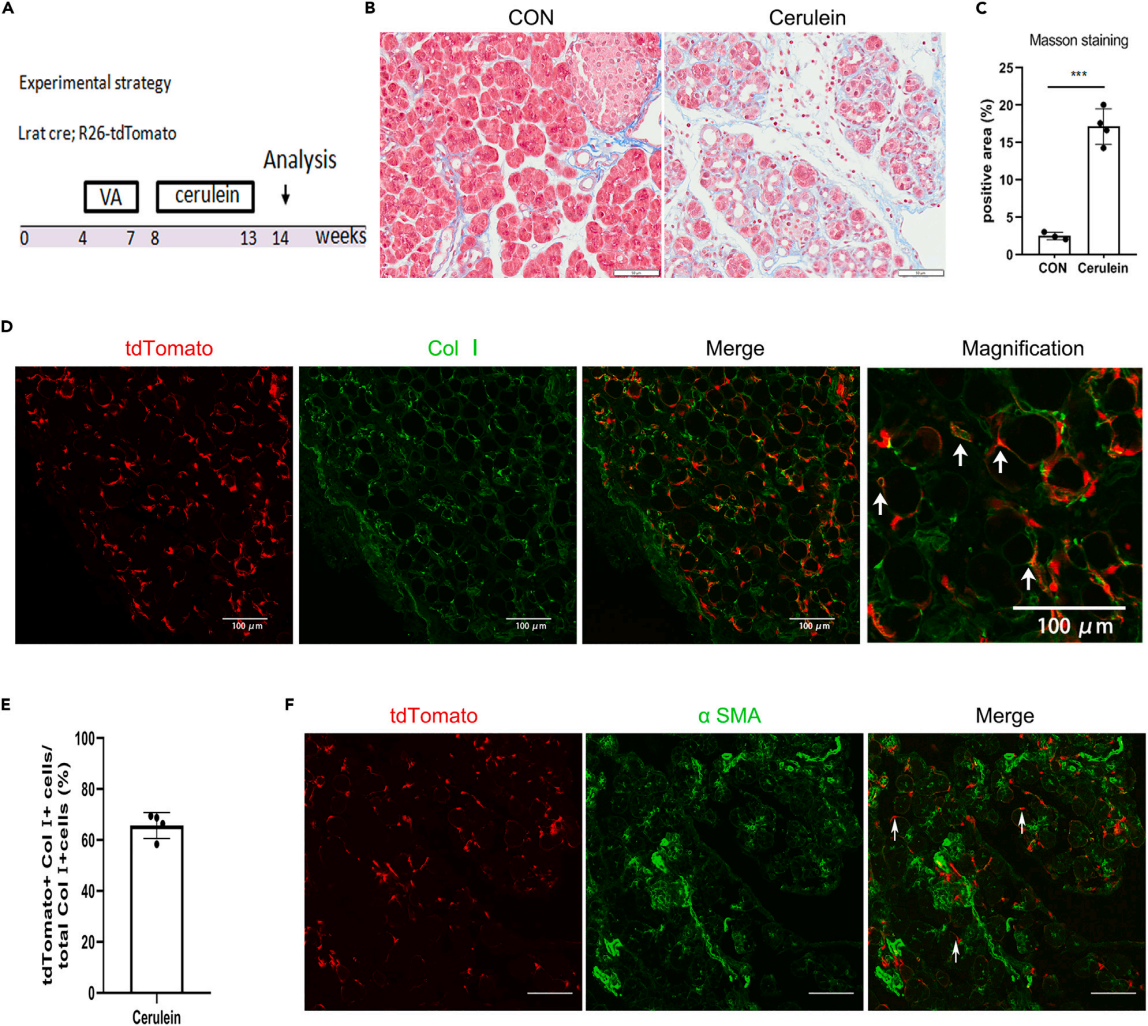
Stellate cells are the major source of myofibroblasts in cerulein-induced pancreatic exocrine fibrosis (A) Schematic diagram illustrating the experimental strategy to test for the contribution of stellate cells to myofibroblasts in pancreatic exocrine fibrosis. Male mice aged 4 weeks were treated with vitamin A palmitate (5000 IU/day for 20 days) and intraperitoneally injected with cerulein (50 μg/kg, six times per day, 3 days/wk) for 6 weeks then euthanized at age of 14 weeks. (B and C) Masson staining of the pancreas from control or cerulein-induced pancreatitis in stellate cell lineage tracing mice and quantification. Fibrotic material in blue and normal myocardium in red. (D and E) Quantification of ColⅠ+ cells derived from Lrat Cre-labelled tdTomato-positive stellate cells was performed in pancreas sections. (F) Co-localization of α SMA with tdTomato in cerulein-induced pancreatic exocrine fibrosis was determined by confocal microscopy. Scale bars, 50 μm (B) and 100 μm (D and F). Data are shown as means ± s.e. ∗∗∗P＜0.001 (determined by Student’s *t* test).

To determine the contribution of stellate cells to cerulein-induced pancreatic exocrine fibrosis, we treated 8-week-old Lrat-cre; R26-tdTomato mice with cerulein for 6 weeks and collected pancreatic tissues at 1 week after injury (Figure 2A). We performed Masson staining and found severe fibrosis in the exocrine tissues of the pancreas (Figures 2B and 2C). Immunostaining for tdTomato and Col Ⅰ on pancreatic sections revealed that 65.7% of collagen-producing cells were tdTomato-positive PSCs (Figures 2D and 2E). Immunostaining for tdTomato and α SMA revealed that tdTomato+ cells express α SMA (Figure 2F). Together, these data indicate that PSCs constitute a major myofibroblast population in cerulein-induced pancreatic exocrine fibrosis.

### The number of stellate cells in islets is increased in high-dose streptozocin-induced T1DM mice

**Figure 3:**
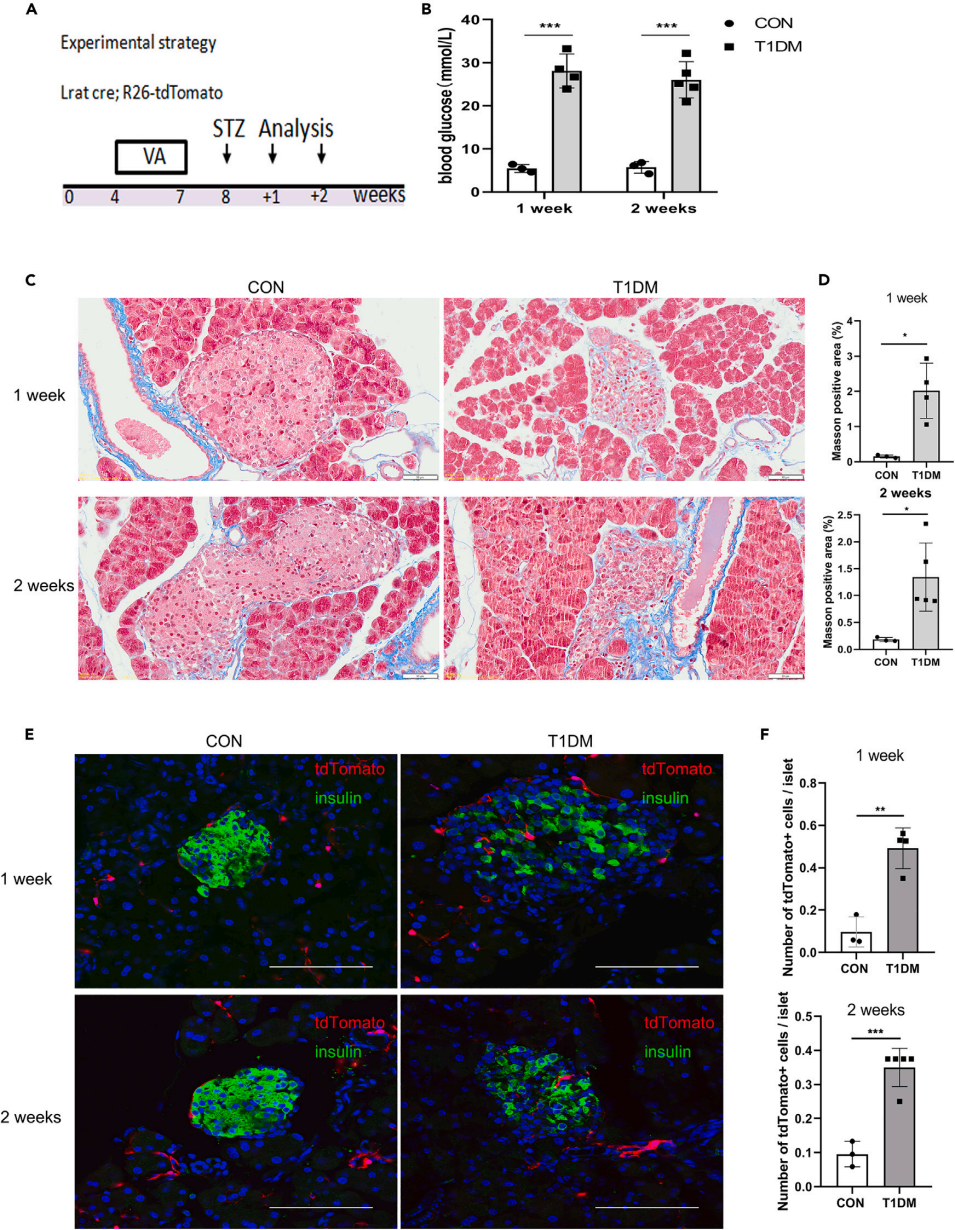
Stellate cells in islets are increased in high-dose streptozocin-induced T1DM (A) Schematic diagram illustrating the experimental strategy to test for the contribution of stellate cells in high-dose streptozocin-induced islet fibrosis. Female mice aged 4 weeks were treated with vitamin A palmitate (5000 IU/day for 20 days) and intraperitoneally injected with one large-dose streptozocin (200 mg/kg) then euthanized at 1 or 2 weeks after injury. (B) Blood glucose levels of mice before euthanization. (C) Masson staining of the pancreas from control or high-dose streptozocin-induced diabetic stellate cells lineage tracing mice. (D) Quantification of the percentage of positive area in Masson staining of the pancreas from control and diabetic mice. (E) Immunostaining for tdTomato and insulin of pancreas collected from control or diabetic mice. (F) Quantification of the numbers of tdTomato+ stellate cells in control and diabetic islets. Scale bars, 50 μm (C) and 100 μm (E). Data are shown as means ± s.e. ∗P＜0.05, ∗∗P＜0.01, ∗∗∗P＜0.001 (determined by Student’s *t* test).

To determine the contribution of stellate cells to pancreatic endocrine islet fibrosis, we treated 8-week-old Lrat-Cre; R26-tdTomato mice with a single high-dose injection of beta-cell specific toxin streptozocin (STZ), followed by analyses at 1 or 2 weeks after injury (Figure 3A). Severe elevation of blood glucose indicated a successful construction of T1DM model (Figure 3B). Next, we performed Masson staining of pancreatic tissue sections and found that islet structural destruction and fibrosis at these two time points (Figures 3C and 3D). Immunohistochemical staining also showed the increased expression of Col1a1, Fn and α-SMA in the islets of diabetic group (Figures S3A and S3B). Immunostaining for tdTomato and insulin (Figures 3E and 3F) or Glucagon (Figures S3C and S3D) revealed an increased number of stellate cells in diabetic islets. Together, these data indicate that stellate cells in islets are increased in high-dose streptozocin-induced islet acute injury and fibrosis.

### The number of stellate cells in islets is increased in HFD and low-dose streptozocin-induced T2DM mice

**Figure 4:**
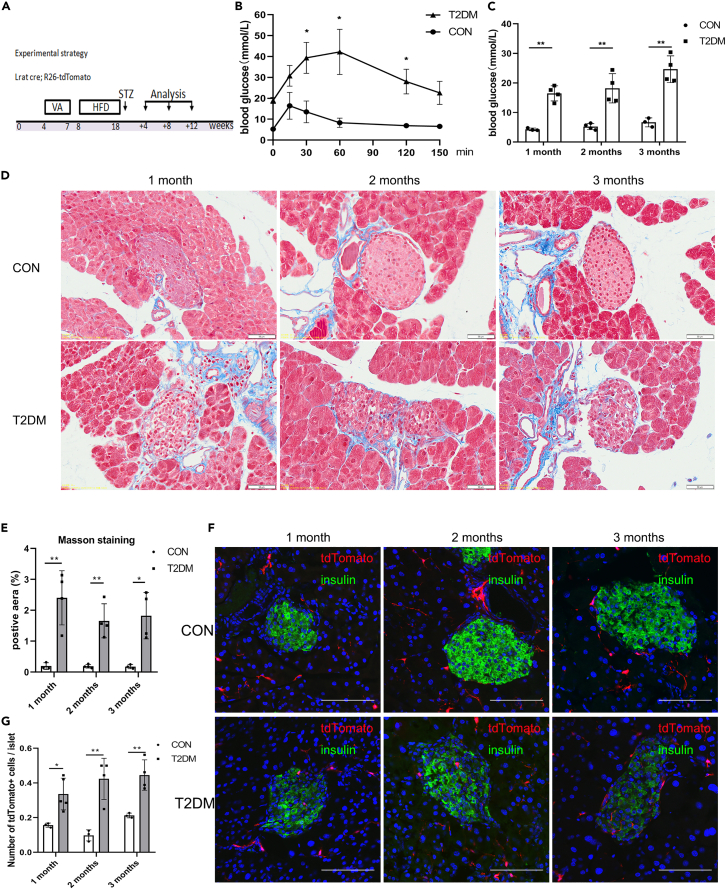
Stellate cells in the islets are increased in HFD and low-dose streptozocin-induced T2DM (A) Schematic diagram illustrating the experimental strategy to test for the contribution of stellate cells in HFD and low-dose streptozocin-induced islet fibrosis. Male mice aged 4 weeks were treated with vitamin A palmitate (5000 IU/day for 20 days), fed a high-fat diet for ten weeks then received multiple low-dose injections of streptozotocin (35 mg/kg/day, 3 consecutive days), and then euthanized at 1, 2 or 3 months after injection. (B) Blood glucose levels of an IPGTT performed in control and T2DM mice. (C) Blood glucose levels of mice before euthanization. (D) Masson staining of pancreas from control or diabetic lineage tracing mice. (E) Quantification of the percentage of positive area in Masson staining of pancreas from control and diabetic mice. (F) Immunostaining for tdTomato and insulin of pancreas collected from control or diabetic mice. (G) Quantification of the numbers of tdTomato+ stellate cells in control and diabetic islets. Scale bars, 50 μm (D), 100 μm (F). Data are shown as means ± s.e. ∗P＜0.05, ∗∗P＜0.01, ∗∗∗P＜0.001 (determined by Student’s *t* test or two-way ANOVA).

After having established the increase of stellate cells in streptozocin-induced islet acute injury and fibrosis, we next determined the contribution of stellate cells to islet chronic injury and fibrosis. We treated 8-week-old Lrat-Cre; R26-tdTomato mice with HFD and low-dose streptozocin, followed by analyses at 1, 2, and 3 months after injury (Figure 4A). Ten-week HFD-feeding and low dose streptozocin made the mice glucose intolerant (Figure 4B) and the blood glucose levels before sacrificing are shown in Figure 4C. Next, we stained pancreatic tissue sections for Masson and observed islet structural destruction and fibrosis at these three time points (Figures 4D and 4E). Immunostaining for tdTomato and insulin (Figures 4F and 4G) or Glucagon (Figures S4A and S4B) revealed that the numbers of stellate cells in diabetic islets were increased. Similar to high-dose streptozocin-induced islet acute injury and fibrosis, these data indicate that stellate cells are increased in the islets of T2DM mice induced by HFD-Fed and low-dose streptozocin.

### Stellate cells in the islets contribute to the myofibroblast pool in both T1DM and T2DM

**Figure 5:**
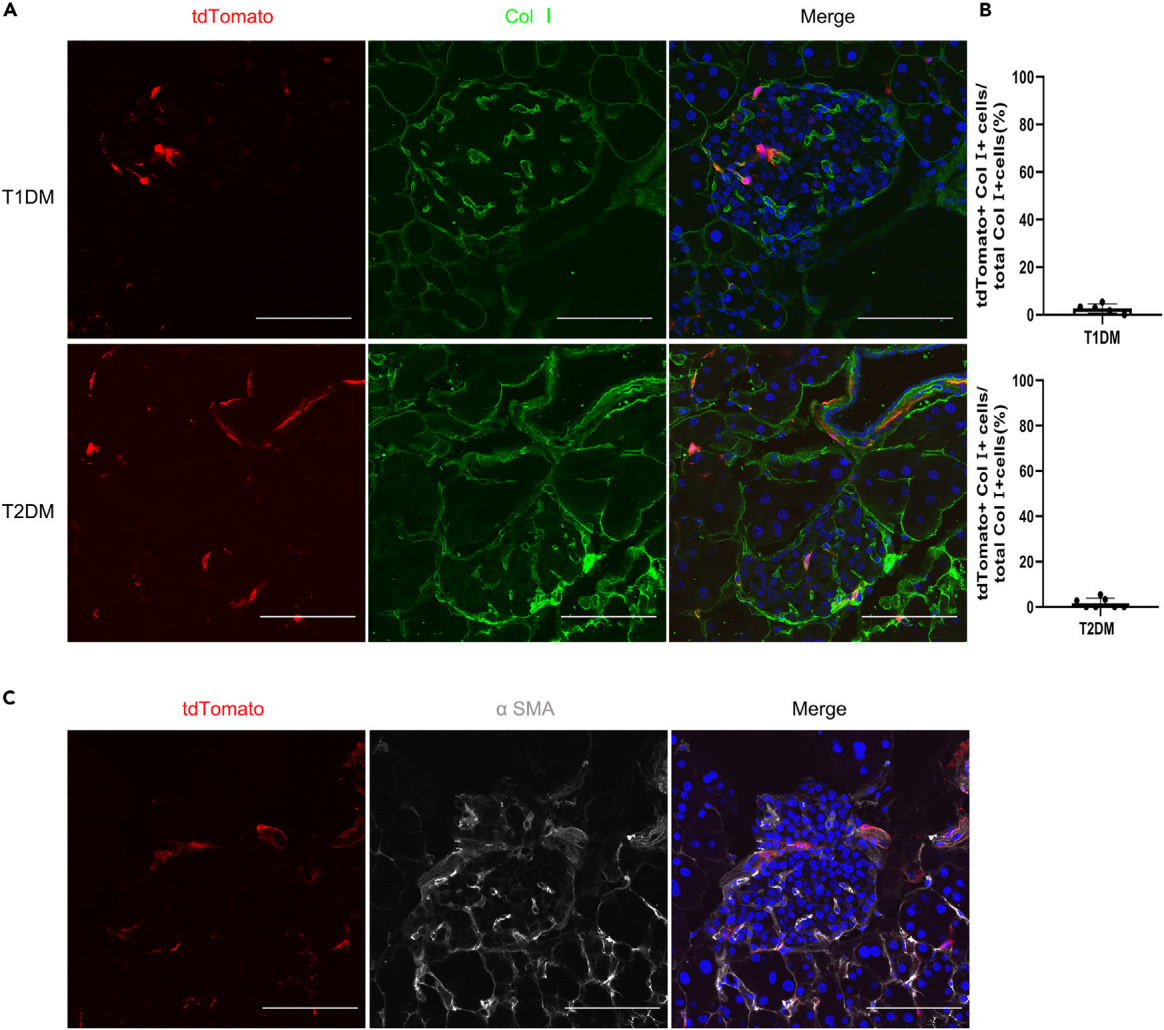
Stellate cells contribute to a small fraction of the myofibroblast pool in the process of islet fibrosis in diabetic mice (A) Co-localization of Col Ⅰ with tdTomato in the islets of T1DM or T2DM determined by confocal microscopy. (B) Quantification of Col Ⅰ+ cells derived from Lrat Cre-labelled tdTomato-positive stellate cells in the islets. (C) Co-localization of α SMA with tdTomato in diabetic islet fibrosis was determined by confocal microscopy. Scale bars, 100 μm. Data are shown as means ± s.e.

After having established the increase of stellate cells in the islets, we next determined the contribution of stellate cells to the myofibroblast pool during islet injury and fibrosis in type 1 or 2 diabetic mice. Immunostaining for tdTomato and Col Ⅰ revealed that most of the stellate cells expressed Col Ⅰ (Figure 5A). However, less than 5% of collagen-producing cells were tdTomato-positive stellate cells (Figure 5B). Immunostaining revealed that stellate cells express α SMA (Figure 5C). Together, these data indicate that stellate cells in the islets constitute a small myofibroblast population in type 1 or 2 diabetic islet fibrosis.

### Pancreatic stellate cells are genetically ablated when mice are injected with vitamin A palmitate

**Figure 6:**
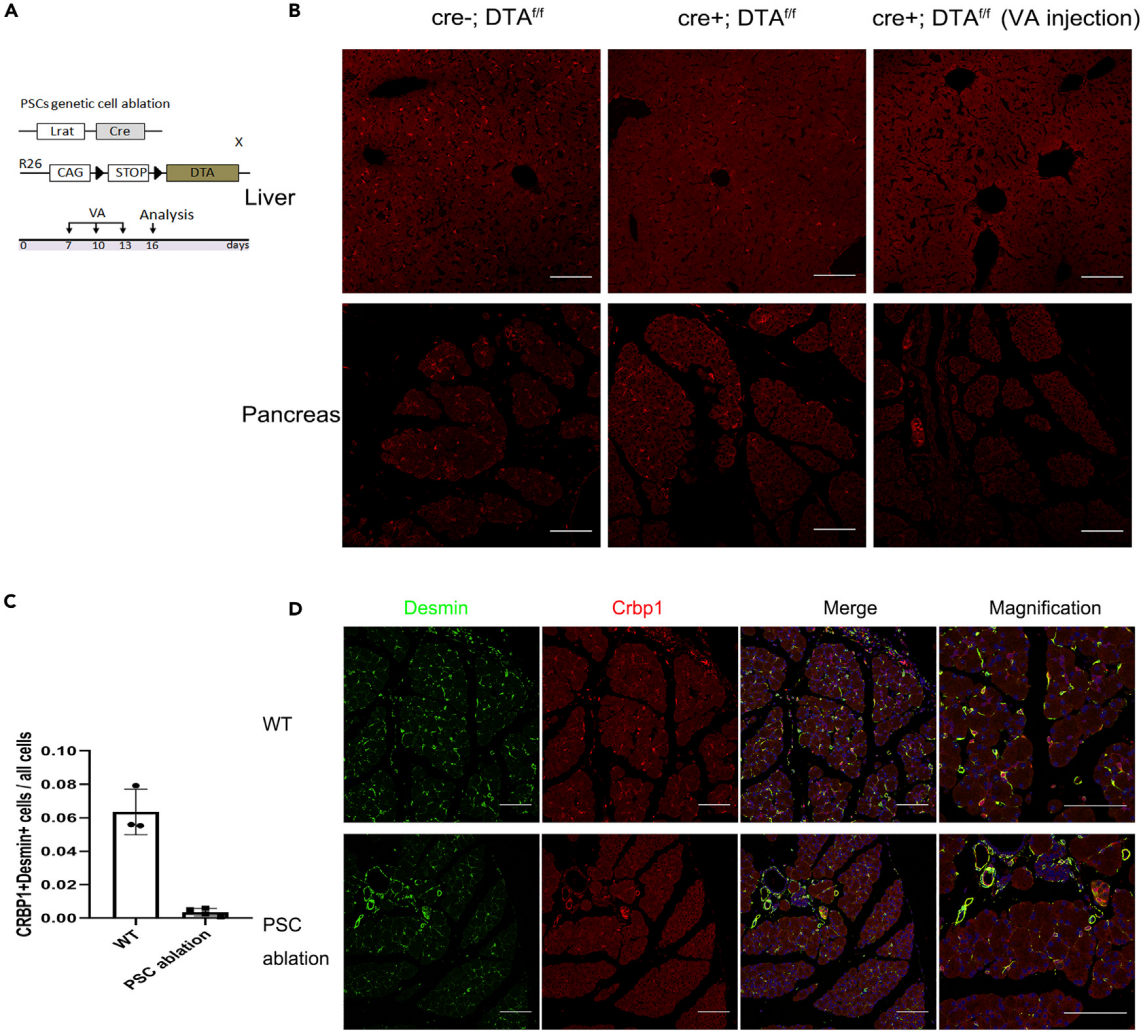
Pancreatic stellate cells are genetically ablated when mice are injected with vitamin A palmitate (A) Schematic diagram illustrating the generation of genetic ablation of stellate cells and the experimental strategy. (B) Immunostaining for Crbp1 in liver and pancreas collected from Control (Cre-; DTA^f/f^), HSCs/PSCs ablation (Cre+; DTA^f/f^) or HSCs/PSCs ablation+ VA mice. (C and D) Immunostaining for Crbp1 and desmin (two stellate cell markers) and quantification of the percentage of Desmin+Crbp1+ cells in the pancreas of WT and pancreatic stellate cell ablation mice. Scale bars, 100 μm (B and D).

To further substantiate the functional contribution of stellate cells to fibrogenesis in pancreatic exocrine and endocrine tissues, we ablated stellate cells via Lrat Cre-induced diphtheria toxin (DTA) in stellate cells (Figure 6A). We found most of hepatic stellate cells were ablated but pancreatic stellate cells were reserved in Lrat Cre; Rosa26-DTA mice (Figure 6B). Then we attempted to eliminate pancreatic stellate cells by intraperitoneal injection of vitamin A (induce the expression of cre-recombinase in pancreatic stellate cells). In addition, results show that vitamin A palmitate injection (2500 IU/time, 3 times in 3 days intervals) strongly reduced stellate cell number determined by immunostaining of stellate cell markers Desmin and Crbp1 in the pancreas of mice treated with vitamin A palmitate (Figures 6C and 6D). Therefore, by vitamin A injection, we developed a pancreatic stellate cells genetically ablated method in the Lrat Cre; Rosa26-DTA mice.

### Stellate cells genetic ablation improves pancreatic exocrine fibrosis but not islet fibrosis

**Figure 7:**
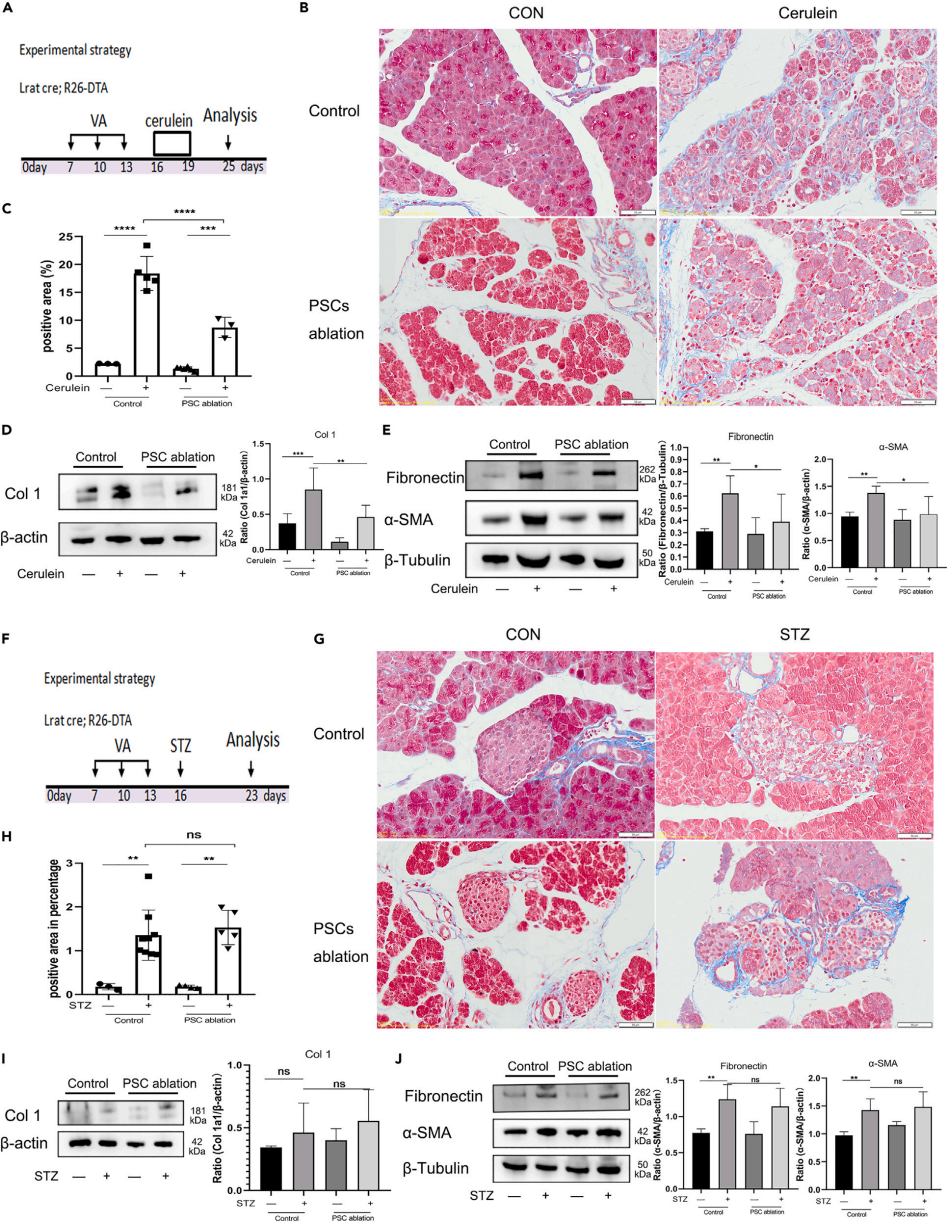
Stellate cell genetic ablation improved pancreatic exocrine fibrosis but not islet fibrosis (A) Schematic diagram illustrating the experimental strategy to test the role of stellate cell ablation in pancreatic exocrine fibrosis. (B) Masson staining of the pancreas from control or cerulein-induced pancreatitis in control and stellate cell ablation mice. (C) Quantification of the percentage of positive area in Masson staining of the pancreas. (D) Western blotting analysis of Col 1protein in pancreas isolated from control (cre-; DTA^f/f^) and PSC ablation (cre+; DTA^f/f^) mice in response to cerulein. (E) Western blotting analysis of fibronectin and α-SMA protein in pancreas isolated from control (cre-; DTA^f/f^) and PSC ablation (cre+; DTA^f/f^) mice in response to cerulein. (F) Schematic diagram illustrating the experimental strategy to test the role of stellate cell ablation in pancreatic islet fibrosis. (G) Masson staining of the pancreas from control or streptozocin-induced diabetes in control and ablation mice. (H) Quantification of the percentage of positive area in Masson staining of the islets. (I) Western blotting analysis of Col 1 protein in pancreas isolated from control (cre-; DTA^f/f^) and PSC ablation (cre+; DTA^f/f^) mice in response to STZ-induced diabetes. (J) Western blotting analysis of fibronectin and α-SMA protein in pancreas isolated from control (cre-; DTA^f/f^) and PSC ablation (cre+; DTA^f/f^) mice in response to STZ-induced diabetes. Scale bars, 50 μm (E and H), 100 μm (B). Data are shown as means ± s.e. ∗P＜0.05, ∗∗P＜0.01, ∗∗∗P＜0.001, ∗∗∗∗P＜0.0001 (determined by one-way ANOVA).

We treated stellate cells ablation mice with cerulein for 3 consecutive days (50 μg/kg, six times per day), and collected pancreatic tissues on day 6 after injury (Figure 7A). Masson staining showed that the area percentage of blue-labeled collagen tissue significantly decreased in stellate cell ablated cerulein-treated mice (Figures 7B and 7C). Western blotting showed that stellate cells ablation decreased cerulein-induced up-expression of Col 1, Fibronectin and α-SMA proteins in pancreatitis ((Figures 7D and 7E). We also treated stellate cells ablation mice with a single STZ injection after 3 times of vitamin A palmitate administration and then analyzed the pancreas samples at 7 days after injection (Figure 7F). But no significantly decreased of the area percentage of blue-labeled collagen tissue in islet of ablated mice treated with STZ as determined by Masson staining (Figures 7G and 7H). Western blotting also showed that no significantly decreased of Col 1, Fibronectin and α-SMA proteins in STZ treated PSC ablation mice compared to WT-DM mice (Figures 7I and 7J). Together, these data indicate that stellate cells ablation improve pancreatic exocrine fibrosis but not islet fibrosis.

## Discussion

In this study, we developed a novel fate-tracing strategy for stellate cells by vitamin A administration. Using chemicals-induced pancreatic exocrine or islet fibrosis models, we found that stellate cells contributed crucially or partly to myofibroblast pools in exocrine or islet fibrosis. Furthermore, stellate cells’ genetic ablation improved pancreatic exocrine fibrosis. These data suggest that stellate cells are vital/partial contributors to pancreatic exocrine/islet fibrosis. Our findings provide solid *in vivo* evidence that stellate cells as important antifibrotic target cells in pancreatic fibrosis.

Because of the lack of specific promoters, there has been no breakthrough in genetic tracing techniques for PSCs *in vivo* in the past decade. The stellate cells can be detected using different markers: those with ectoderm origin [e.g., GFAP, nestin]; mesoderm origin [desmin, α-SMA]; and vitamin A related metabolic markers [e.g., LRAT, Crbp1].^21^^,^^22^ However, previous studies have shown that the ectoderm origin promoters, like GFAP, do not efficiently mark HSCs,^4^ and that the mesoderm origin promoters are also expressed in other mesenchymal cells (like pericytes).^23^^,^^24^ Therefore, in this study, we tried to put Cre under the mice Lrat promoter because this strategy has been successfully applied to HSCs lineage tracing.^4^ Surprisingly, only a small fraction of PSCs was labeled with tdTomato by this strategy in physiological conditions. This may be because of the fact that PSCs rarely store vitamin A in normal dietary mice.^20^ Therefore, we tried to improve the labeling efficiency based on vitamin A anabolic function of LRAT protein.^18^^,^^19^ We up-regulated the transcription of Lrat gene, thereby increasing Cre expression by Vitamin A loading and the results were consistent with our expectations.

PSCs are generally recognized as the pancreatic counterpart cells of HSCs. Despite the lack of solid *in vivo* evidence that PSCs are the primary drivers of pancreatic exocrine fibrosis, much of the current research focuses on this cell type. Our results are in line with previous studies suggesting that targeted interventions on PSCs can improve pancreatic exocrine fibrosis.^25^^,^^26^^,^^27^^,^^28^ Recently, Gen Yamamoto found that only a minority (21.8%) of collagen-producing cells were GFAP-positive. Then he concluded that myofibroblasts in the pancreas might be derived not only from PSCs but also from other fibrogenic cells.^20^ However, this may be because of the fact that GFAP expression is nonexistent or significantly reduced in activated PSCs.^29^^,^^30^ Our data also suggests that a subset of myofibroblasts is not derived from PSCs. As reported in the previous study, bone marrow-derived fibrocytes may contribute to pancreatic exocrine fibrosis.^31^^,^^32^ In addition, the pericytes, abundant but long-neglected cells in the pancreas, similar to their role in fibrotic diseases in other tissues, may also differentiate into myofibroblasts.^33^^,^^34^^,^^35^ In recent years, it has been found that endothelium can transdifferentiate into myofibroblasts through endothelial-to-mesenchymal transition and participate in organ fibrosis.^36^^,^^37^ Therefore, more research is needed to identify those possible cell types in the future, such as cell lineage tracing and bone marrow transplantation experiment.

For endocrine islets, the primary cells responsible for fibrosis are unclear, but previous studies have suggested multiple cell types implicated in islet fibrogenesis. By genetic tracing, Mateus Gonçalves L found that islet pericytes contribute a substantial fraction (approximately 40%) of islet myofibroblasts in AktTg mice (a mouse model of islet vascular fibrosis).^6^ Melvin R Hayden found that pericyte can differentiate into an islet pancreatic stellate–myofibroblast-like cell capable of synthesizing fibrillar-banded collagen in the islet-exocrine interface.^38^^,^^39^ Advanced glycation end products have also been shown to induce islet endothelial cells endothelial-to-mesenchymal transition *in vitro*, which may contribute to islet fibrosis in diabetes.^40^ We previously isolated stellate cells from islets and found that they were involved in the process of islet fibrosis.^41^^,^^42^ Consistent with a previous study.^12^ our data suggest that stellate cells are increased in islets of type 1 or 2 diabetes and contribute to a small subset of myofibroblasts in the process of islet fibrosis.

Taken together, our results show that stellate cells are mainly distributed in the pancreatic exocrine while a small portion was observed in the endocrine islets. Stellate cells are vital contributors to the myofibroblast pool in cerulein-induced pancreatic exocrine fibrosis, and genetic ablation of stellate cells can improve pancreatic exocrine fibrosis. Besides, during the process of islet fibrosis induced by streptozocin, stellate cells in the islets are increased and partly contribute to the myofibroblast pool. So, we conclude that stellate cells are vital/partial contributors to myofibroblasts in pancreatic exocrine/islet fibrosis. Our study provides the *in vivo* lineage tracing evidence for the cellular target in pancreatic fibrosis.

### Limitations of the study

Our study has several limitations. First, the loads of vitamin A may affect the activation of PSCs. Several studies have shown that vitamin A inhibits the activation of PSCs or HSCs.^43^^,^^44^^,^^45^ However, a study also showed that the absence of retinoid-containing lipid droplets (Lrat KO mice) does not promote HSCs activation.^46^ In this study, after a 10-day gavage of vitamin A, the labeling efficiency of PSCs did not significantly improve. This may be because of the fact that vitamin A is primarily stored in HSCs but not PSCs.^47^ In addition, the labeling efficiency rapidly increased from 15- to 18-day and maintained after 20-day gavage of vitamin A. Furthermore, the undetectable vitamin A autofluorescence in PSCs of the tracing mice implied a low intracellular amount of vitamin A, which is similar to the negative results in normal dietary mice in a previous study.^20^ So, we speculate that the PSCs in this strategy may not be very different from physiological conditions. Second, because of lifetime limitation, 2–4 weeks ablated mice were used to substantiate the functional contribution of PSCs to fibrogenesis. This is not representative of the adult stage, and superior ablation mouse models are needed in further studies. Third, we only adopted chemicals-induced fibrosis models, which may not be applicable to other pathological conditions. Fourth, as reported in previous studies, bone marrow derived cells contribute to pancreatic fibrosis. In addition, the role of BM cells in pancreatic fibrosis will be more fully verified if further bone marrow transplantation experiments are performed. Besides, pericytes and endothelium may also be involved in pancreatic fibrosis. So related cell tracing and ablation experiments can more completely and clearly show the cellular mechanism of pancreatic fibrosis, which is our follow-up research direction of pancreatic fibrosis research. Finally, our results provide solid *in vivo* evidence that stellate cells as important antifibrotic target cells in pancreatic fibrosis, but PSC ablation is unlikely to be a clinical application directly because of the other physiological functions of PSC (such as vitamin A storage and metabolism). So, compare to PSC ablation, future development of new drugs or inhibitors targeting PSC may be more beneficial to the clinical prevention and treatment of pancreatic fibrosis. In this study, we also mainly focused on fibrosis itself, and further functional tests will be conducted in future studies to better provide experimental evidence for clinical prevention and treatment.

## STAR★Methods

### Key resources table

**Table:** 

REAGENT or RESOURCE	SOURCE	IDENTIFIER
**Antibodies**		

anti-Desmin	Abcam	Cat# ab32362, RRID: AB_731901
anti-PDGFRb	Thermo Fisher Scientific	Cat# 14-1402-81, RRID: AB_467492
anti-PDGFRb	CST	Cat# 3169, RRID: AB_2162497
anti-Ki67	Abcam	Cat# ab15580, RRID: AB_443209
anti-α-SMA	Abcam	Cat# ab32575, RRID: AB_722538
anti-α-SMA	Proteintech	Cat# 14395-1-AP, RRID: AB_2223009
anti-Collagen I	Abcam	Cat# ab34710, RRID: AB_731684
anti-Collagen I	Abcam	Cat# ab6308, RRID: AB_305411
anti-Fibronectin	Proteintech	Cat# 15613-1-AP, RRID: AB_2105691
anti-Insulin	Bioss	Cat# bs-0056R, RRID: AB_10883837
anti-Glucagon	Bioss	Cat# bs-3796R, RRID: AB_10857341
anti-CRBP1	Santa	Cat# sc-271208, RRID: AB_10610075
Goat Anti-Rabbit IgG H&L (Alexa Fluor® 647)	Abcam	Cat# ab150079, RRID: AB_2722623
Goat Anti-Rabbit IgG H&L (Alexa Fluor® 488)	ZSGB-BIO	Cat# ZF-0511, RRID: AB_2864279
Goat Anti-Mouse IgG H&L (Alexa Fluor® 594)	ZSGB-BIO	Cat# ZF-0513, RRID: AB_2892725
Goat Anti-Rat IgG H&L (FITC)	Servicebio	GB22302
anti-β-Tubulin	Fude technology	FD0064
anti-β-Actin	Proteintech	Cat# 66009-1-Ig, RRID: AB_2687938

**Chemicals, peptides, and recombinant proteins**		

Vitamin A palmitate	Aladdin	Cat# R106319
Cerulein	MedChemExpress	Cat# HY-A0190
Streptozocin	Solarbio	Cat# S8050

**Experimental models: Organisms/strains**		

Mouse: Lrat cre: B6/JGpt-Lrat^em1Cin(P2A-iCre)^/Gpt	Gempharmatech	Strain # T006205
Mouse: Rosa26-tdTomato: C57BL/6JSmoc-Gt (ROSA)26Sor^em (CAG-LSL-tdTomato)1Smoc^	Shanghai Model Organisms	Stock # NM-KI-225042
Mouse: Rosa26-DTA: B6.129P2-Gt (ROSA)26Sor^tm1(DTA)Lky^/J	Jackson laboratory	Strain # 009669

**Oligonucleotides**		

Primer: lrat Forward: TCAACAGGCACTGGAGCCTTG	This paper	N/A
Primer: lrat Reverse: ATCCTTGGCACCATAGATCAGGC	This paper	N/A

**Software and algorithms**		

Image J	NIH	N/A
Image pro plus	Media Cybernetics	N/A
GraphPad Prism (v8)	GraphPad Software	N/A

### Resource availability

#### Lead contact

Further information on resources and reagents should be directed to the lead contact, Dr. Zilin Sun (sunzilin1963@126.com).

#### Materials availability

This study did not generate new unique reagents. And mouse lines generated in this study are availablefrom the lead contact, Dr. Zilin Sun (sunzilin1963@126.com).

### Experimental model and subject details

#### Materials

Vitamin A palmitate (CAS NO. 79-81-2, Aladdin, China) was dissolved in corn oil and introduced by gavage or intraperitoneal injection. Cerulein (Stock NO. HY-A0190) was purchased from MedChemExpress, USA. Streptozocin (Code NO. S8050) was obtained from Solarbio, China.

#### Mice

Lrat cre mice (Strain NO. T006205) were generated using the CRISPR–Cas9 technology by Gempharmatech (Jiangsu, China). Briefly, a cDNA encoding Cre was targeted to the translational start codon ATG of the lrat gene by homologous recombination. PCR primer were designed to test the correctly targeted allele (lrat cre,5′mut-F: TCAACAGGCACTGGAGCCTTG, 5′mut-R: ATCCTTGGCACCATAGATCAGGC).Rosa26-loxp-Stop-loxp-tdTomato (Stock NO. NM-KI-225042) mice were purchased from Shanghai Model Organisms. Rosa26-loxp-Stop-loxp-DTA mice with C57BL/6 background were kindly provided by Prof. Bo O. Zhou of the Chinese Academy of Sciences, Shanghai. For lineage tracing experiments, Rosa26-tdTomato mice were crossed with Lrat cre recombinase mice. And cre-positive tdTomato-positive mice were given VA palmitate (5000 IU/day, 20 days) by gavage from the fourth week. Eight-week mice were used for pancreatic or islet fibrosis model construction. For genetic cell ablation experiments, Rosa26-DTA mice were crossed with Lrat cre recombinase mice. And cre-positive DTA-positive mice were intraperitoneally administered vitamin A palmitate (2500 IU/time, 3 times in 3 days intervals) from the seventh day. Approximately 16-day-old mice were used for ablation experiments. All mice were housed in standard cages and maintained on a 12-h light/dark cycle. This study was approved by the Institutional Animal Care and Use Committee of Southeast University (Date 2020.03/ No. 202003200006).

#### Pancreatic or islet fibrosis models

Pancreatic exocrine fibrosis was induced by intraperitoneal injection of cerulein (50μg/kg, six times per day, 3 d/wk) for 6 weeks or 3 consecutive days (50 μg/kg, six times per day) in 8-week male mice. Control male mice received an equal amount of sterile normal saline. Endocrine islet fibrosis was induced by intraperitoneal injection of streptozocin (STZ) in two conditions: Eight-week chow-fed female mice were exposed to a single high-dose of STZ (or vehicle control) to resemble acute islet injury of type 1 diabetes mellitus; And 8-week male mice fed a high-fat diet for ten weeks then received multiple low-dose injections of streptozotocin (35mg/kg/day, 3 consecutive days) to emulate the chronic islet fibrosis of T2DM. Chow-fed mice injected with vehicle buffer were used as controls for T2DM groups.

### Method details

#### Vitamin A autofluorescence

To detect the autofluorescence of vitamin A, parts of the livers or pancreas were quickly cut and fixed by paraformaldehyde for 2 hours in total darkness, and 20-μm thick sections were made with a freezing microtome. The sections were examined with Olympus confocal microscopy system (excitation filter BP365/12, barrier filter BP495/40) for the detection of the rapidly fading blue autofluorescence that is characteristic of vitamin A.

#### Histological examination

Formalin-fixed, paraffin-embedded specimens were sectioned at a 4-μm thickness and were stained with Masson Stain Kit (G1006, Servicebio, China) following manufacturer protocol. All imaging data were acquired using a slide scanning system (Olympus VS200), and the positive or total areas were calculated using Image Pro Plus software.

#### Immunofluorescence

For immunofluorescence (frozen), the specimens were fixed with 4% paraformaldehyde for 4-6 hours, then cryoprotected in 30% sucrose/PBS at 4°C overnight and followed by embedding in optimal cutting temperature compound. Afterward, 10 μm thickness slices were collected and blocked for 30 min at room temperature. For immunofluorescence (Paraffin), the specimens were dehydrated and embedded in paraffin. Then 4 μm tissue sections were cut and dewaxed. Heat-mediated antigen retrieval was performed in TE buffer pH 9.0. The sections were incubated with a primary antibody at 4°C overnight, followed by a secondary antibody at room temperature for 1 hour, and then counterstained with DAPI. After slides were mounted with fluorescence-protecting mounting medium (S2100, Solarbio, China), images were obtained with a confocal microscopy system (FV1000, Olympus, Japan). The antibodies used for this were: anti-Desmin (Abcam ab32362), anti-PDGFRb (Thermo 14-1402-81), anti-PDGFRb (CST 3169), anti-Ki67 (Abcam ab15580), anti-α-SMA (Abcam ab32575), anti-Collagen I (Abcam ab34710), anti-Insulin (Bioss bs-0056R), anti-Glucagon (Bioss bs-3796R), anti-CRBP1 (Santa sc-271208), Goat Anti-Rabbit IgG H&L (Alexa Fluor® 647) (Abcam ab150079), Goat Anti-Rabbit IgG H&L (Alexa Fluor® 488)( ZSGB-BIO zs-0511), Goat Anti-Mouse IgG H&L (Alexa Fluor® 594)(ZSGB-BIO zs-0513), Goat Anti-Rat IgG H&L (FITC)( Servicebio GB22302).

#### Western blotting

Tissues were grinded in a homogenizer (KZ-III, Servicebio, China) and proteins were isolated with RIPA lysis buffer (Beyotime Biotech, China) supplemented with protease inhibitors (Servicebio, China). After quantification, the proteins were subjected to SDS-PAGE electrophoresis and immediately transferred to a polyvinylidene fluoride membrane (Millipore, CA, USA). the blots were blocked with 5% skim milk and then incubated with primary antibodies at 4°C overnight. The primary antibodies used for this were: anti-α-SMA (Proteintech 14395-1-AP), anti-Collagen I (Abcam ab6308), anti-Fibronectin (Proteintech 15613-1-AP), anti-β-Tubulin (Fude technology FD0064) and anti-β-Actin (Proteintech 66009-1-Ig). Next, goat anti-rabbit or anti-mouse antibodies (Biosharp Biotech; 1:10000) were then applied, and immunoreactivity was detected using enhanced chemiluminescence (Fude technology, China) and imaged with Chemiluminescence Imager system. Quantitative analysis was performed via Image J software.

### Quantification and statistical analysis

Experiments were performed on at least three mice per treatment group. Desmin, PDGFRb, or tdTomato-positive cells were counted in merged 200- or 400-fold pictures. To determine Col Ⅰ positive cells originating from stellate cells, at least 8 random 200-fold pictures per mouse were analyzed. To determine the number of tdTomato-positive cells in diabetic or control islets, at least 15 random 200-fold islet pictures per mouse were analyzed. Data are expressed as mean ± SD or mean ± SEM from at least 3 animals per group. GraphPad Prism (v8) was used for statistical analyses. Unpaired, two-tailed Student’s t-tests were performed to analyze the p value for single comparisons between two groups and one-way ANOVA with Tukey’s post hoc tests or two-way ANOVA with Bonferronior post hoc tests were used to determine significant differences between multiple groups. Significance was accepted when < 0.05.

### Additional resources

No other additional resources.

## Data Availability

All data presented in this study will be shared by the lead contact Dr. Zilin Sun (sunzilin1963@126.com) upon request. This paper does not report any original code. Additional information required to reanalyze the data reported in this paper is available from the lead contact upon request.
