# Supplementary material for: Genetic lineage tracing reveals stellate cells as contributors to myofibroblasts in pancreas and islet fibrosis
Source: iScience. 2023 May 26;26(6):106988. doi: 10.1016/j.isci.2023.106988 (PMC10291507; doi:10.1016/j.isci.2023.106988)
Supplement: Document S1. Figures S1–S4 [file mmc1.pdf]

iScience, Volume *26*

Supplemental information

Genetic lineage tracing reveals stellate cells as contributors to myofibroblasts in pancreas and islet fibrosis

Jinbang Wang, Tingting Li, Yunting Zhou, Xiaohang Wang, Vladmir Carvalho, Chengming Ni, Wei Li, Qianqian Wang, Yang Chen, Zhanjia Shang, Shanhu Qiu, and Zilin Sun

Supplemental information

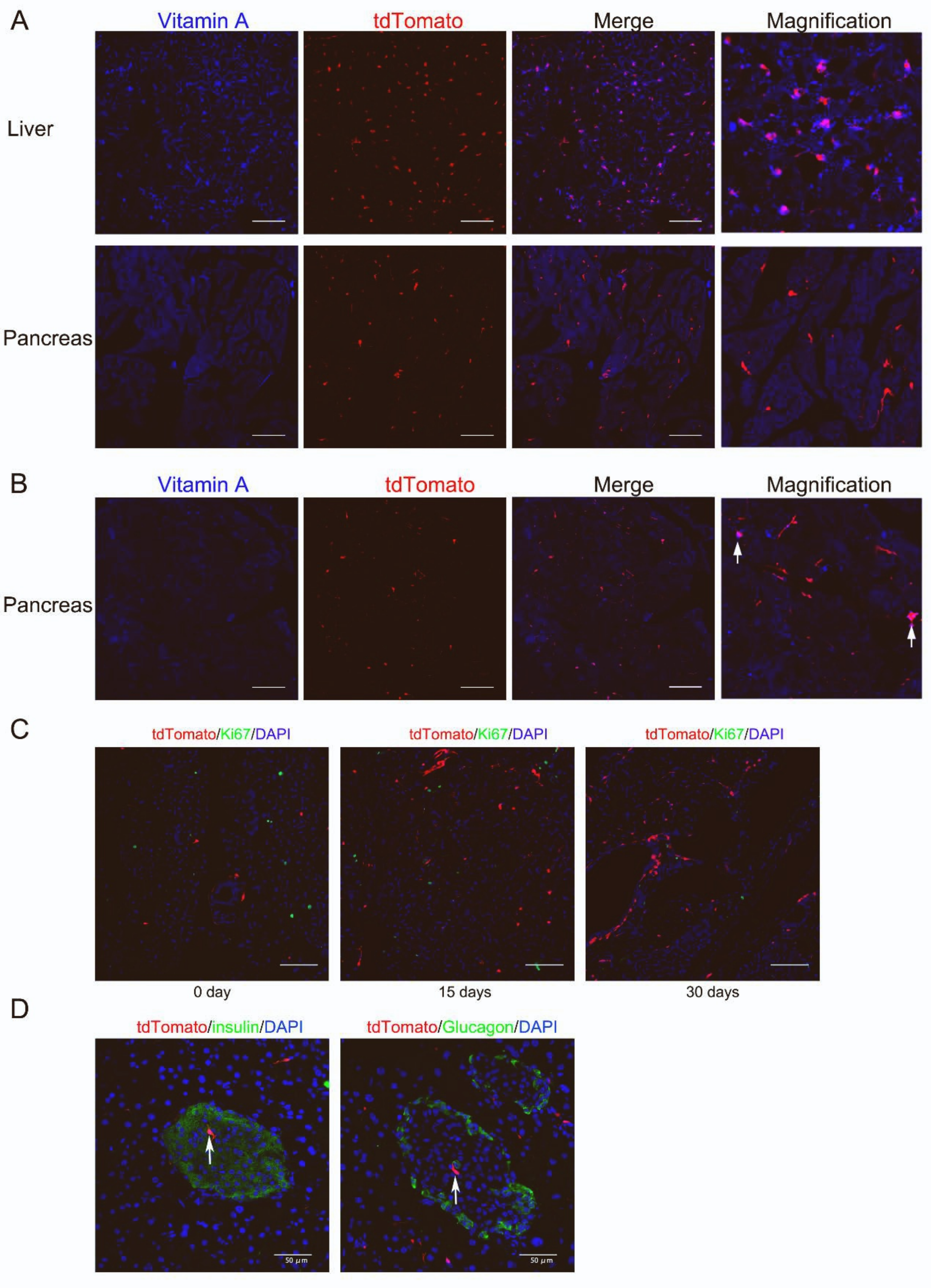

Figure S1 | Identification of tdTomato+ cells. Related to Figure 1. A, Co-localization of Lrat Cre induced tdTomato expression and vitamin A autofluorescence in the liver and pancreas of mice without vitamin a palmitate gavage. **B,** Co-localization of Lrat Cre induced tdTomato and vitamin A autofluorescence in the pancreas of mice with vitamin a palmitate (5000 IU/day, 40 days) gavage. **C,** Co-localization of Lrat Cre induced tdTomato with ki67 in

the pancreas of mice treated with vitamin a palmitate at 0, 15 and 30 days. d, Immunostaining for tdTomato and Insulin or Glucagon in pancreas. Scale bars 100 μm.

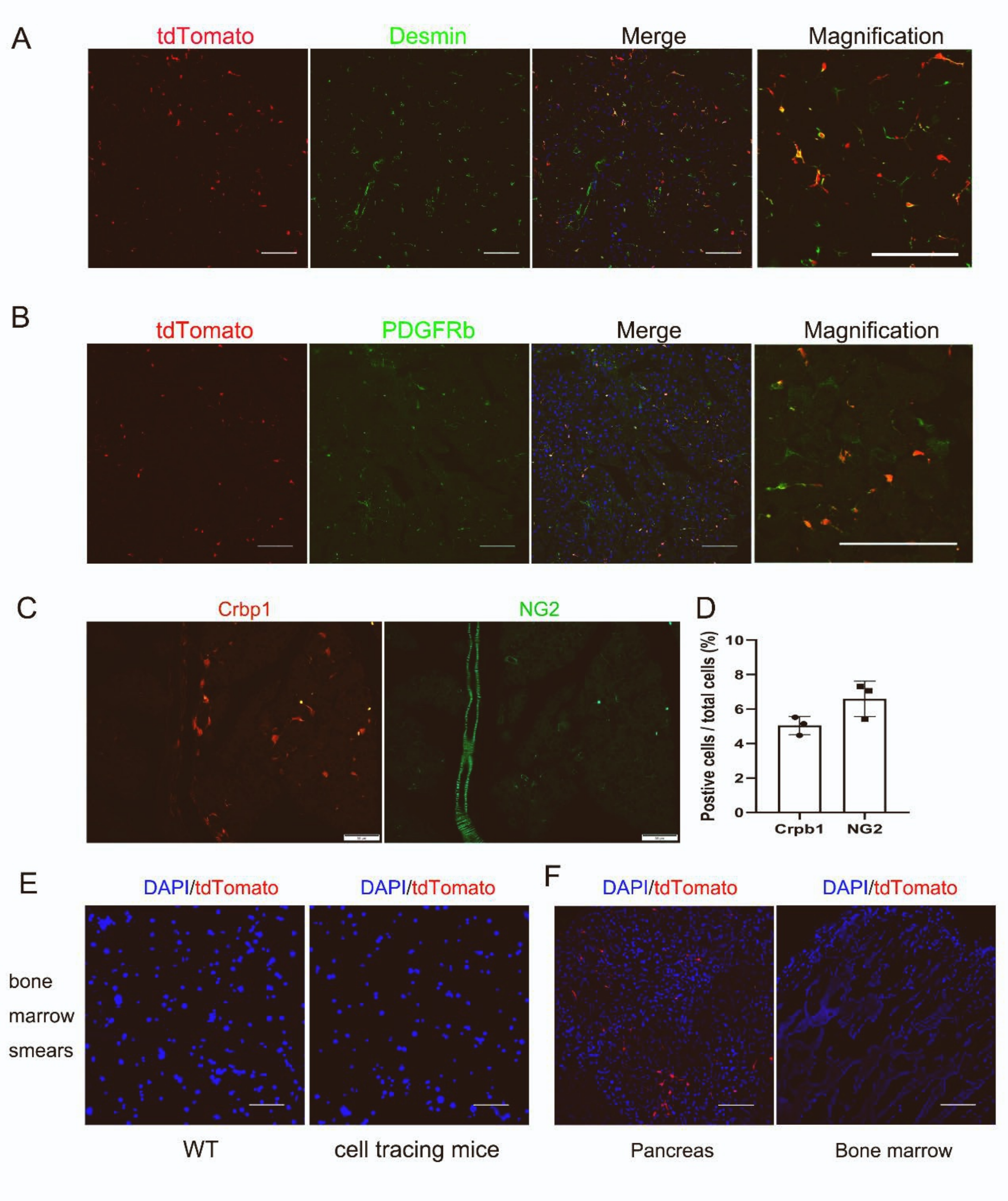

Figure S2 | tdTomato+ cells are pancreatic stellate cells. Related to Figure 1. A, B, Colocalization of Lrat Cre induced tdTomato with Desmin or PDGFRb was determined by immunofluorescence and confocal microscopy. **C, D,** Immunostaining and quantification for Crbp1+ cells (stellate cells) and NG2+ cells (pericytes) in the pancreas. **E,** No tdTomato+ cell in bone marrow smears of WT or PSC cell tracing mouse. **F,** tdTomato+ cell in pancreas but not in bone marrow of PSC cell tracing mice. Scale bars 100 μm.

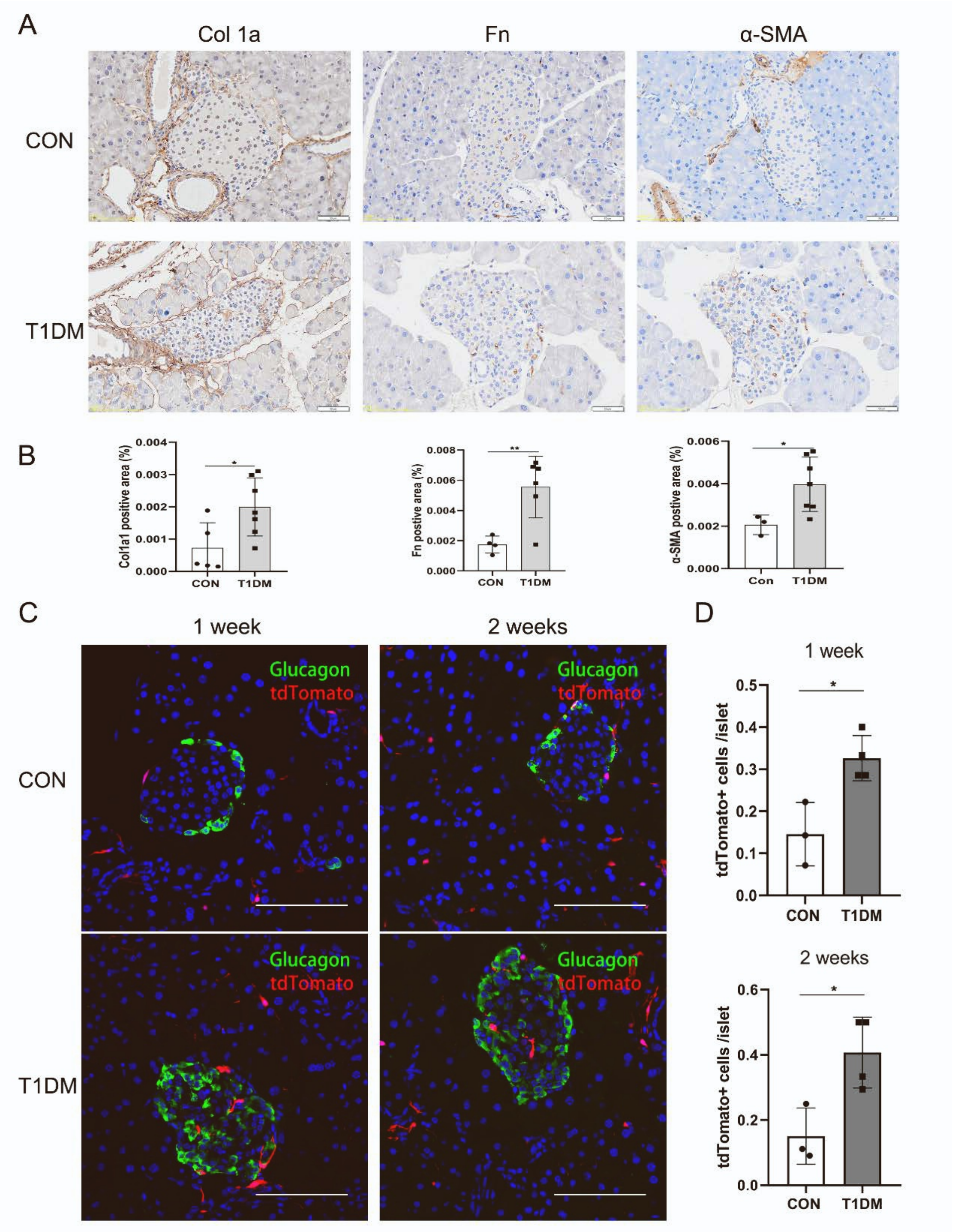

Figure S3 | Stellate cells in islets are increased in T1DM. Related to Figure 3. A, B, Immunohistochemical staining and quantification for Col1a1, Fn and α-SMA in pancreas of control or diabetic mouse. Scale bars 50 μm. **C, D,** Immunostaining and quantification for tdTomato and Glucagon in pancreas collected from control or type 1 diabetic mice. Scale bars 100 μm.

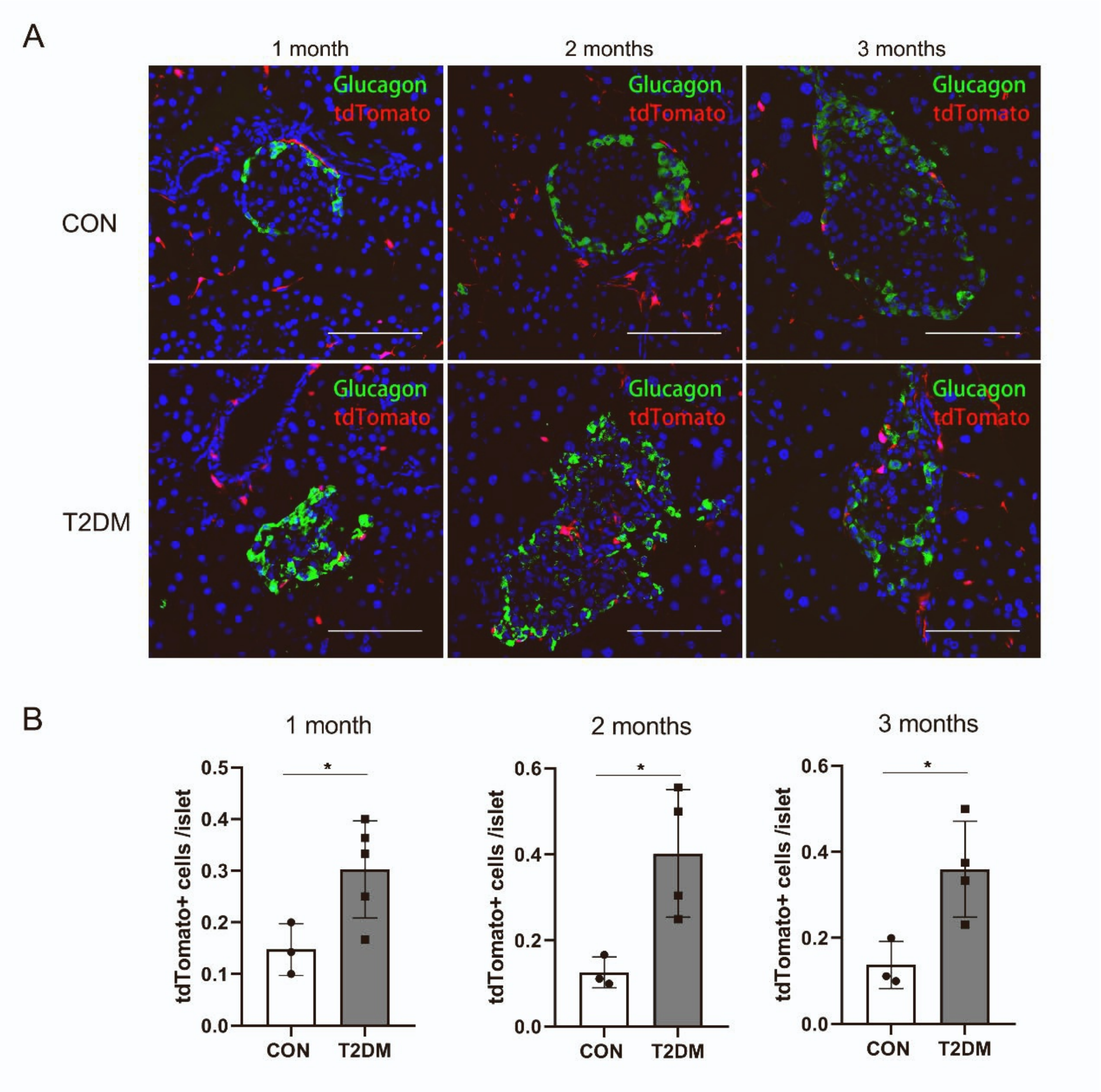

Figure S4 | Stellate cells in islets are increased in T2DM. Related to Figure 4. A, B, Immunostaining and quantification for tdTomato and Glucagon in pancreas collected from control or type 2 diabetic mice. Scale bars 100 µm.
